# Policy implementation deviation of government purchase of old age care services in Jiangsu, China: based on empirical and policy analysis

**DOI:** 10.1186/s12961-024-01108-8

**Published:** 2024-02-15

**Authors:** Hongli Chen, Yue Zhao, Hongxin Huang, Ying Xing, Yueheng Yin, Enfang Shan, Daoxiang Cheng, Yanjian Sun, Xianwen Li

**Affiliations:** 1 School of Nursing, Nanjing Medical University, Nanjing, 211166 China; 2 The First Clinical Medical College, Nanjing Medical University, Nanjing, 211166 China; 3 Red Cross Society of Jiangsu Branch, Nanjing, 210029 China

**Keywords:** Government purchase, Old age care, Policy analysis, Implementation deviation

## Abstract

**Background:**

Government purchase of social forces to participate in old age care services can release the burden of social care. Current research on performance evaluation in this field mainly focussed on the establishment of appropriate evaluation indices. However, discussion on the policy implementation deviation is scarce. This study aimed to evaluate the performance of China’s local government purchase of old age care services, analyse the characteristics of related policies and explore their deviation.

**Methods:**

The persons who participated in the Training of the Trainer (ToT) organized by the Red Cross Society were enrolled. The policy documents were obtained from the official websites. The *K*-means cluster was used to determine the project performance grades. We compared the project performance grades between service objects and undertakers with different characteristics utilizing the non-parametric test. Based on the framework of ‘Collaborative Participation – Project Performance Objective’, we analysed the content of relevant policy aiding by NVivo 12.

**Results:**

Data of project performance were collected from 306 participants. The standardized mean score of the efficiency dimension was the lowest (0.70 ± 0.24). The projects were divided into four grades: poor (17.0%), average (27.5%), good (12.4%) and excellent (43.1%). There were statistically significant differences in project performance grades only between advanced ageing groups (*Z* = 2.429, *P* = 0.015). As well, the policy also mentioned that the services focus should be tilted towards the oldest old. The purchasers mainly involved the Ministry of Civil Affairs and Health management departments in the policy. Respite services were less mentioned in the responsibilities of the undertakers. The requirement for efficiency and effectiveness was mentioned in less than half of the policy documents.

**Conclusion:**

Policy attention is needed for the responsibilities and functions of the intermediate purchasing force, as well as more precise directions and responsibilities of undertakers. The purchasers and undertakers should improve management abilities and capacity of old age care services and focus on associated factors to achieve the best marginal benefit. In addition, the embedded performance evaluation needs to be updated periodically to bridge the deviation between policy implementation and policy formulation.

**Supplementary Information:**

The online version contains supplementary material available at 10.1186/s12961-024-01108-8.

## Background

As the largest developing country, China experienced significant declining fertility rates and accelerating population ageing, with a current adults aged over 65 years old of around 209.78 million, accounting for 14.9% of the total population by 2022 [1]. Compared with developed countries, the situation of ‘getting old before getting rich’ makes the country suffer multiple pressures in professional human resources and family caregivers for old age care [2]. To tackle the issues of high operating costs of old age care [3], policymakers have grown interested in the government’s purchase of public services. Government purchase of services involves transferring some public services, which are currently provided by the government, to qualified social forces under government supervision. Previous studies on the participation of social forces in old age care services show that social care forces can make up for the shortage of professional caregivers and reduce their pressure [4, 5]. The research regarding the investment and value evaluation of the government purchase of old age care services also revealed that the social forces played an important role in supplementing the gap in the professional old age care team [6]. At the same time, in the context of the reforms to ‘streamline administration, delegate powers and improve regulation and services’ in China, the position of social forces has become more prominent and important in public governance. To give full play to the role of social care forces in dealing with the ageing society, the government of China has issued a series of policies aimed at speeding up the supply-side structural reform of old age care services. As one of the four pilot provinces in China, Jiangsu province has explored the government purchase of social forces to carry out Training of the Trainer (ToT) and old age care services since 2017, named the Senior Care Volunteers Training Program (SCVTP).

At the beginning of SCVTP, the methods of performance evaluation mainly focused on a single dimension such as the dimension of the older adults or the undertakers, while ignoring the multidimensional performance of the project. In addition, current research on the performance evaluation of government purchase of old age care services mainly focussed on the establishment of appropriate evaluation indicators [7]. However, there is a scarcity of research that discussed issues from the perspective of policy implementation deviation between policy formulation and project implementation. Policy implementation deviation refers to the gap between government’s aspiration and policy implementation. It is mainly characterized by inconsistency with the policy content, deviating from the policy objectives, and so on [8]. In the Smith Policy Implementation Model, the implementation of policies can be hindered by various factors (e.g., policy itself, the skills and capabilities of the policy implementation agencies, and the external environment), resulting in the distortion, deformation or reduction of effectiveness of the policies, deviating from their intended goal [9]. Most articles discussed policy implementation deviation by conducting opinion research [10]. In the field of long-term care and rural mutual assistance for older adults, several researchers explored these issues by combining the methods of case analysis or cross-sectional study and policy analysis [11, 12], which provides some useful insights for designing our research. Nevertheless, there is still a lack of robust evidence and research regarding policy implementation deviation in government purchase of old age care services. This may lead to the failure to achieve the expected performance objectives of the projects proposed in the policy and mismatch between policy and practice. Therefore, it is essential to carry out the performance evaluation on the implementation of government purchase of old age care services, combined with analysing the policy document of related topic, through which we can find the deviation between them. At the same time, the discussion on the project performance difference among service objects with different characteristics such as regions, ageing and advanced ageing groups, as well as different professional undertakers, will be helpful to clarify the characteristics of the performance grade distribution. It will provide a basis for the scientific fund investment management of the project for government and provide a starting point for the optimization of existing policies in this field.

Taking Red Cross Society of Jiangsu Branch as an example, we aimed to evaluate the old age care service projects purchased by government using the evaluation index system [13] developed by the research group in the early stage. At the same time, based on a framework matching the variables of performance, undertakers and service objects in the quantitative empirical study, we conducted the content analysis of the national-level policies related to the government purchase of old age care services. Through these, we expect to discuss the current performance grade of the empirical project, the content of relevant policies and their deviation, which could provide a basis for the improvement of the performance grades of the government purchase of old age care service projects and the optimization of supporting policies in the future.

## Methods

### Research design

A multi-method research including cross-sectional design and policy document analysis was utilized to explore deviation between policy and implementation in terms of government purchase of social forces to participate in old age care services. Multi-method research relies on triangulation to merge the results obtained through different methods, which is suitable for our need [14, 15]. The projects purchased by the Red Cross Society of Jiangsu Branch were enrolled to analyse the implementation performance. As well, the policy documents in the related field in China were analysed to clarify expected policy objectives. Policy analysis enables the exploration of ideas and frames deployed by policy actors during the policy development process. Policy document analysis is a research method for systematically analysing the contents of policy documents outlined by Cardno [16]. It contains two different ways of analysing: inductively and deductively. Our focus was on the purpose of how the policy was constructed and issues related to its implementation. To achieve this, pre-determined categories are essential. We applied the latter. In the deductive approach, very often a model or theory established is the basis for content analysis. Thus, two key steps of the deductive content analysis were concluded: (1) setting an analysis framework and (2) document coding.

### Data collection

#### Data of performance evaluation

The persons who participated in the old age care ToT organized by the Red Cross Society of China Jiangsu Branch were investigated. Purposive sampling was used to select participants who met the inclusion and exclusion criteria. Inclusion criteria included: (1) persons who participated in the ToT from 2017 to 2019 and undertook old age care services purchased by the Red Cross Society of Jiangsu Branch were included; (2) the trainers should establish or expand old age care services more than 6 months; and (3) they volunteered to participate in the questionnaire survey and signed the electronic informed consent. Exclusion criteria included: (1) participants who failed to complete the purchase service as required; (2) participants who spent less than 2 s filling out each item of the questionnaire [17]; (3) they self-report that they did not answer the questionnaire carefully [18]; and (4) the data was incomplete or did not meet the analysis requirements. Cluster analysis was used in this study to classify performance grades. Previous study explicitly mentioned that there is no clear sample size estimation method for continuous variables in the cluster analysis when the number of clusters is unknown. Finally, 306 samples (one participant excluded due to the incomplete questionnaire) were included in the study which met the sample size requirements of cluster analysis (10*d*k, with *d* representing the number of segmentation variables and k representing the number of clusters or segments. In the present study *d* = 6 and *k* = 4) [19].

The data of performance evaluation were collected online. We carried out the self-evaluation of the old age care service projects. The variables of the training years, professions, and regions of the trainers were derived from the questionnaire. Considering the accessibility of the data of service objects, the data of ageing and advanced ageing were derived from the *Report on the Information of the Aging Population and the Development of the Aging Industry in Jiangsu Province* [20].

#### Data of policy documents

The inclusion criteria of the policy documents are as follows: (1) the content of the policy must explicitly mention the performance objective of the government purchase of social forces to participate in the old age care services, directly specify or reflect the relevant content; (2) policy types included planning, opinions, notifications, schemes, implementation plans, laws, regulations and other documents reflecting government policies; and (3) considering that the empirical study on government purchase of old age care service projects started from 2017, the policy documents published at the national level in the past 5 years (from 1 January 2017 to 31 December 2021) were included. Exclusion criteria included: (1) news reports, conference speeches, work reports and documents interpreting policies in this field; and (2) policy documents that duplicate in the included policy document data pool.

The policy documents were obtained from the official websites of The State Council and its ministries and commissions, as well as the PKULAW Database, which is the largest Chinese policy full-text database compiling public policy documents promulgated in China since 1949. In addition, to supplement our results, we retrieved the documents in the existing pension information platform. The search keywords were ‘government purchase of old age care services’ and ‘government outsourcing of old age care services’. In total, 23 policies in this field were retrieved, shown in Additional file 1.

### Instruments

#### Performance evaluation questionnaire

The old age care services evaluation questionnaire developed by the research group was used to evaluate the performance of the China's local government purchase of old age care services. It includes 45 items in six dimensions (relevance, efficiency, effectiveness, satisfaction, social impact and sustainability), and each item has a corresponding weight coefficient [13]. Each item was scored according to Likert level 10; the higher the score, the more consistent it is with the description of this item. In this study, the Cronbach’s *α* coefficient of this questionnaire was 0.979, indicating good internal consistency. At the same time, the questionnaire also contained sociodemographic information about the undertakers’ professions, training years and regions.

#### Policy document analysis framework

**Fig. 1:**
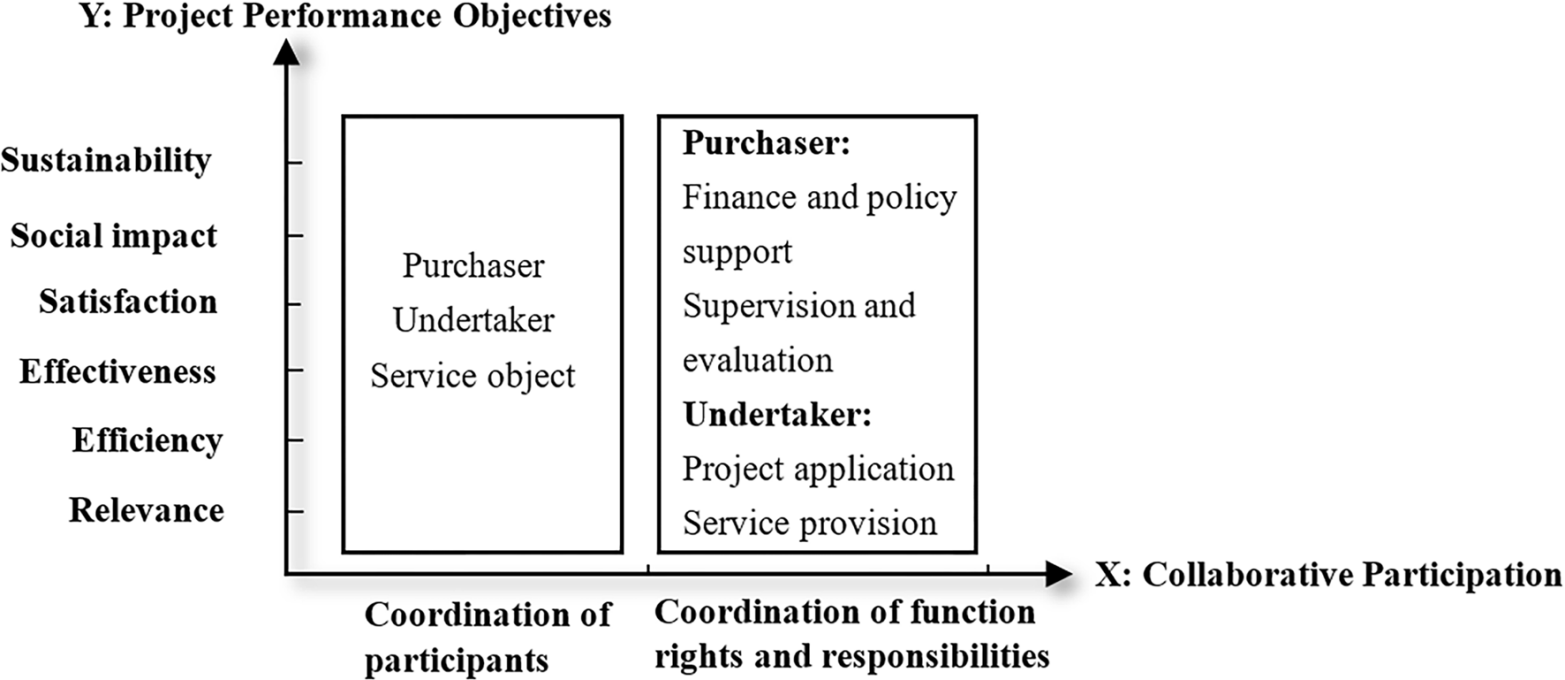
A two-dimensional framework for analysing the policy regarding government purchase of old age care services

The framework of policy document analysis was formulated on the basis of the Liu’s research results of the synergistic relationship between government departments and social forces [21], matching the variables in quantitative empirical research. Based on the framework, we expected to explore the deviation between theoretical policy formulation and actual project implementation. The two-dimensional analysis framework of ‘Collaborative Participation – Project Performance Objectives’ is shown in Fig. 1, with (1) *X* axis represents Collaborative Participation. It is composed of two aspects: the coordination of participants and the coordination of function rights and responsibilities of the participants. The setting of coordination of participants aimed to clarify the characteristics of the purchaser, undertaker and service object in the government purchase of old age care services. The coordination of functional rights and responsibilities was established to determine the specific responsibilities of different participants in the process of government purchase of old age care services. The responsibilities of the purchasers include finance and policy support, supervision and evaluation, determination of purchase content, etc. The responsibilities of undertakers contain project application, service provision, etc. (2) The *Y* axis represents Project Performance Objectives. It aims to clarify whether the policy documents reflect the project performance objectives. We set the analysis framework of this dimension according to the dimension of the old age care services evaluation questionnaire. It includes relevance (whether the policy indicates the requirement to meet needs of the older adults and the rationality of the implementation of the project by social organizations), efficiency (time and cost effectiveness and the degree of project experience promotion), effectiveness (implementation effect of the project, service effect for the older adults and their families, etc.), satisfaction (satisfaction with the project among older adults and their families), social impact (impact of the project on the target service objects, social organizations themself and society) and sustainability (sustainability of the development of the project and social organization).

### Statistical analysis

IBM® SPSS® Statistics software (Armonk, New York, USA) was used to analyse the quantitative data. Firstly, *K*-Means cluster analysis was carried out for each dimension of the evaluation questionnaire. The sum of the squared errors (SSE) and Silhouette Coefficient (*S*) were calculated by Python software as indicators for selecting the best cluster [22]. To verify the rationality of the discriminant classification, the discriminant function was established applied with Fisher multi-class Discriminant analysis method [23]. Additionally, the non-parametric test was conducted to examine the project performance grade differences between the service objects with different regions, ageing degrees and advanced ageing degrees, as well as undertakers with different training years and professions (*α* = 0.05). In the policy document analysis, we deductively encoded the contents conforming to the dimensions of the analysis framework aiding by NVivo 12. Codes was described with frequency distributions. The Gephi 0.9.3 was employed to visualize the cooperative relationship between policymaking departments. In addition, the results of cross-sectional study and policy analysis were compared based on the analysis framework to identify the relationship or deviation between policy design and implementation.

## Results

### Empirical analysis results about performance evaluation of China’s local government purchase of old age services

#### The score of project performance

There are weight differences among all dimensions. To make a better comparison between all dimensions, the weight score difference was standardized. The score of efficiency is the lowest (0.70 ± 0.24). See Table 1 for details.

**Table 1:** Standardized scores of each dimension of project performance

Items	Deviation standardized score	Actual range	Original range
Project performance	0.78 ± 0.19	0.00–1.00	0.00–1.00
Relevance	0.80 ± 0.22	0.00–1.00	0.00–1.00
Efficiency	0.70 ± 0.24	0.00–0.98	0.00–1.00
Effectiveness	0.78 ± 0.19	0.00–1.00	0.00–1.00
Satisfaction	0.83 ± 0.20	0.00–1.00	0.00–1.00
Social impact	0.81 ± 0.17	0.01–1.00	0.00–1.00
Sustainability	0.80 ± 0.19	0.00–1.00	0.00–1.00

#### Grade division of project performance

(1) Determination of optimal clustering number (*K* value) of each dimension.

**Fig. 2:**
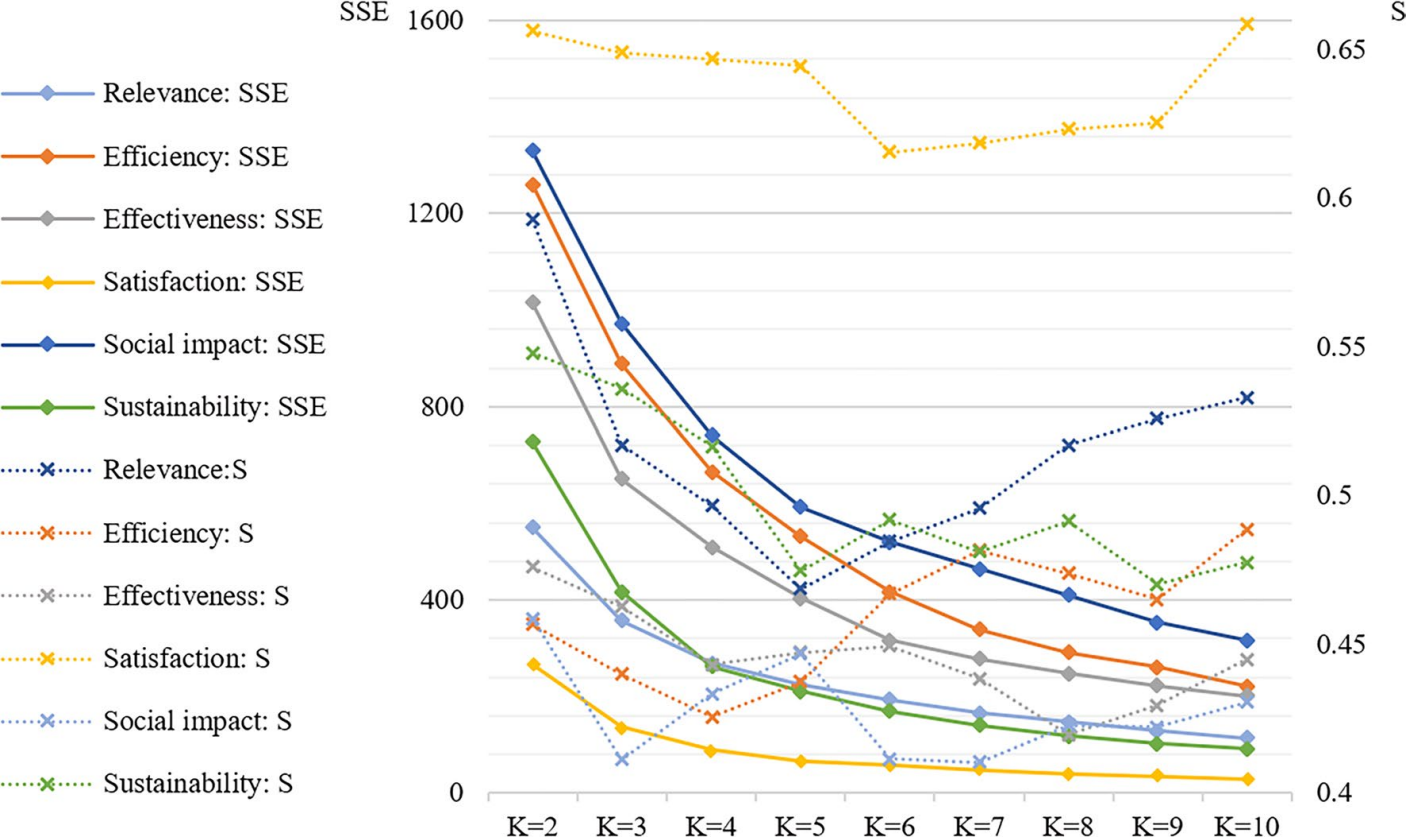
Values for the SSE and *S* in different *K* clusters (SSE: sum of the squared errors, *S*: Silhouette Coefficient)

When *K* does not reach the optimal numbers of cluster, the degree of aggregation of each cluster will greatly increase with the increase of the K value. Hence, the SSE value decreases greatly. After the optimal clustering *K* value is reached, the SSE decreases gently with the increase of the *K* value. The *K* value at the inflection point is the optimal numbers of cluster. The larger the *S* value in the corresponding cluster, the higher the priority. Therefore, after discussion by the research group and considering the practical significance of the grade cluster combining with the value of SSE and *S*, three clusters of each dimension were determined (Fig. 2).

(2) Project performance grade distribution and results of discriminant analysis.

Based on the clustering of each dimension, the overall project performance was clustered again. Considering the performance score and each dimension category of each group comprehensively, four grades of poor (6.57 ± 1.02), average (8.16 ± 0.45), good (8.31 ± 0.89),and excellent (9.56 ± 0.42) were created. In addition, according to the results of cluster analysis, the discriminant analysis was carried out. The discriminant function was established to cross-verify the project performance grades by the leave-one-out classification method. The results revealed that the correct discriminant rate of the four grades was higher, indicating the cluster was reasonable (Table 2).

**Table 2:** Cross validation of the project performance grades (*n*/%)

Performance grades	Score (mean ± standard deviation)	Predicted group membership				Total
		Poor	Average	Good	Excellent	
Poor (52/17.0)	6.57 ± 1.02	48/92.3	3/5.8	1/1.9	0	52 (100)
Average (84/27.5)	8.16 ± 0.45	0	84/100	0	0	84 (100)
Good (38/12.4)	8.31 ± 0.89	0	0	37/97.4	1/2.6	38 (100)
Excellent (132/43.1)	9.56 ± 0.42	0	2/1.5	6/4.5	124/93.9	132 (100)

#### Comparison of project performance grades between service objects and undertakers with different characteristics

According to the economic features of different cities, the cities of Jiangsu were divided into three regions: southern regions, central regions and northern regions [24]. The performance grades between different regions showed no statistical significance. In addition, considering the proportion of younger old and oldest old in different regions and the reasonable distribution of the number of cities [20], the ageing and the advanced ageing were divided into three intervals as shown in Table 3. The project performance grades between different advanced ageing groups had statistical significance (*Z* = 2.429, *P* = 0.015), while there was no statistical significance among different ageing groups. Moreover, no statistical significance was found in the project performance grades between different training years and professions of undertaker (Table 3).

**Table 3:** Comparison of performance grades between various service object and undertaker groups (*n*/%)

Characteristics (*n*)			Grades				Z/*P*-value
			Poor	Average	Good	Excellent	
Service object	Region	Southern regions (108)	15/13.9	33/30.6	14/13.0	46/42.6	-0.328/0.743
		Central regions (58)	12/20.7	13/22.4	6/10.3	27/46.5	
		Northern regions (140)	25/17.9	38/27.1	18/12.9	59/42.1	
	Ageing group	15% ~ < 20% (74)	11/14.9	23/31.1	6/8.1	34/45.9	0.005/0.996
		20% ~ < 25% (100)	16/16.0	30/30.0	15/15.0	39/39.0	
		25% ~ 30% (132)	25/18.9	31/23.5	17/12.9	59/44.7	
	Advanced ageing group	13% ~ < 14% (112)	23/20.5	28/25.0	22/19.6	39/34.8	**2.429/0.015**
		14% ~ < 15% (104)	20/19.2	32/30.8	9/8.7	43/41.3	
		15% ~ 18% (90)	9/10.0	24/26.7	7/7.8	50/55.6	
Undertaker	Profession	Doctors/preventive medicine workers (37)	3/8.1	12/32.4	4/10.8	18/48.6	-1.502/0.133
		Nurses (161)	29/18.0	40/24.8	21/13.0	71/44.1	
		Social workers (70)	10/14.3	19/27.1	10/14.3	31/44.3	
		Others (38)	10/26.3	13/34.2	3/7.9	12/31.6	
	Training year	2017 (69)	13/18.8	16/23.2	14/20.3	26/37.7	1.431/0.152
		2018 (92)	14/15.2	35/38.0	10/10.9	33/35.9	
		2019 (145)	25/17.2	33/22.8	14/9.7	73/50.3	

### Results of the policy document analysis of the government purchase of old age care service performance

#### Characteristics of policy documents

**Fig. 3:**
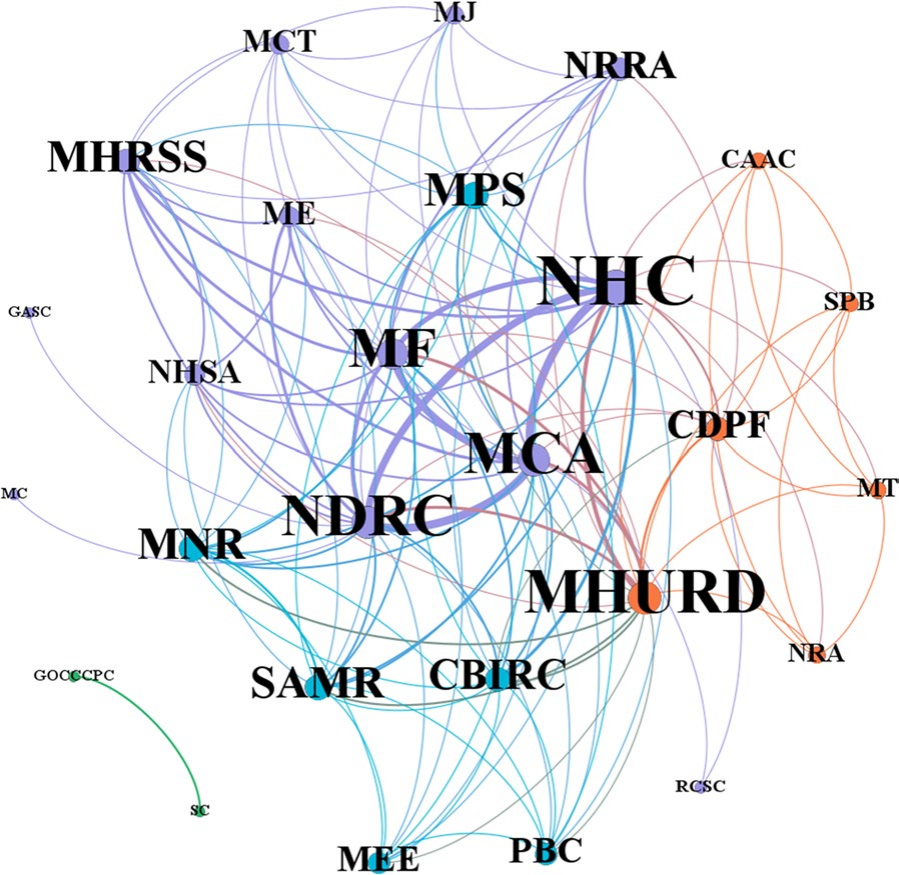
Corporation network among different policymaking departments

Five types of policy documents were retrieved containing opinions (11, 48%), notifications (5, 22%), planning (3, 13%), implementation plans (3, 13%) and schemes (1, 4%). The 29 cooperative subjects constituted 138 cooperative edges. The larger the font and node of the policymaking department name, the more extensive the cooperation between this department and other departments. And the thicker the edges among departments, the more frequent the cooperation among them. The National Health Commission (NHC), the National Development and Reform Commission (NDRC), the Ministry of Civil Affairs (MCA), the Ministry of Finance (MF) and the Ministry of Housing and Urban–Rural Development (MJURD) had more cooperation with other departments. The former four policymaking departments cooperate relatively frequently (Fig. 3).

#### Contents analysis of policy documents based on the two-dimensional framework

**Fig. 4:**
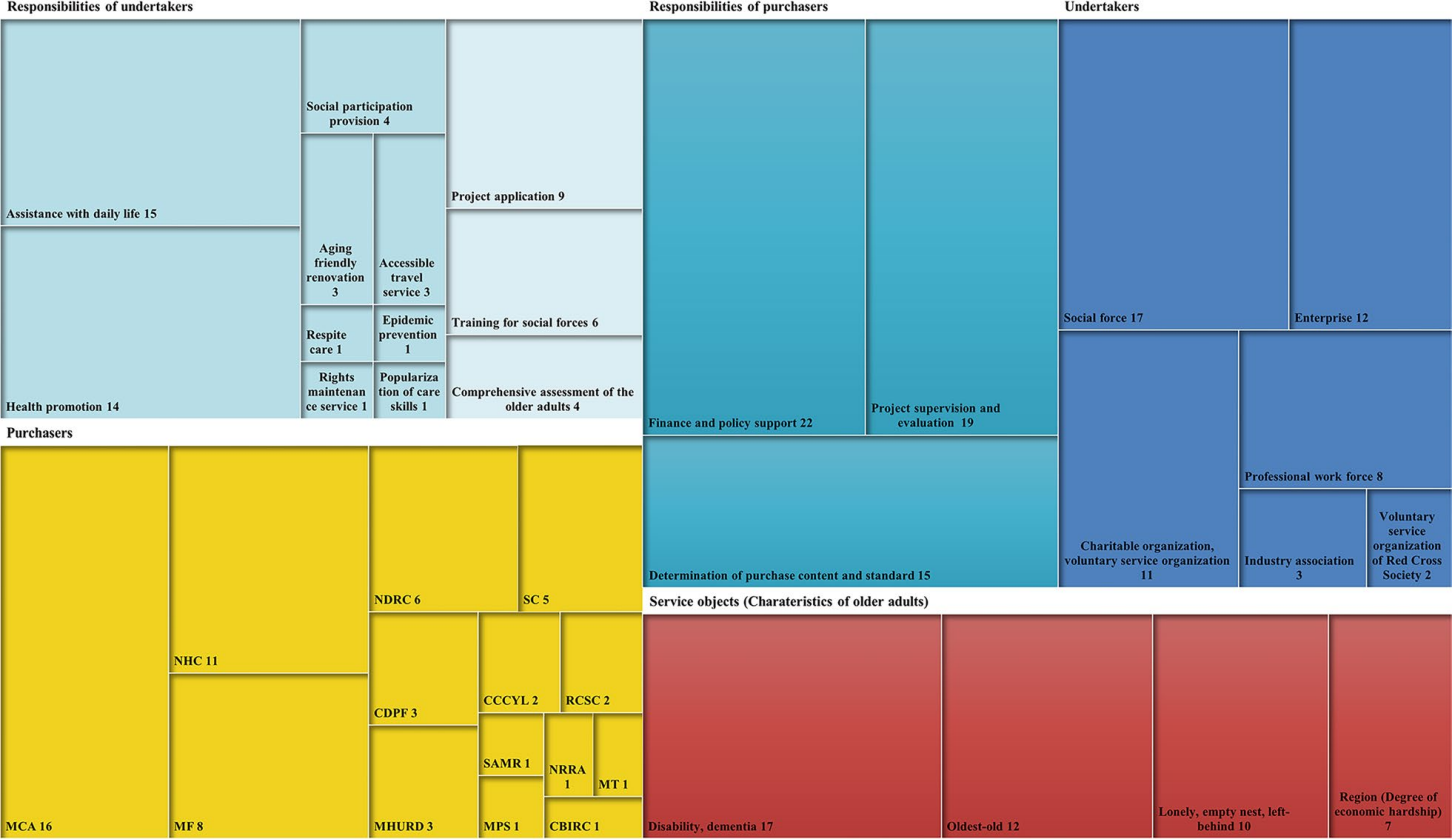
Tree diagram of ‘coordinated participation’ from the policy documents (*n* = number of policy documents)

**Fig. 5:**
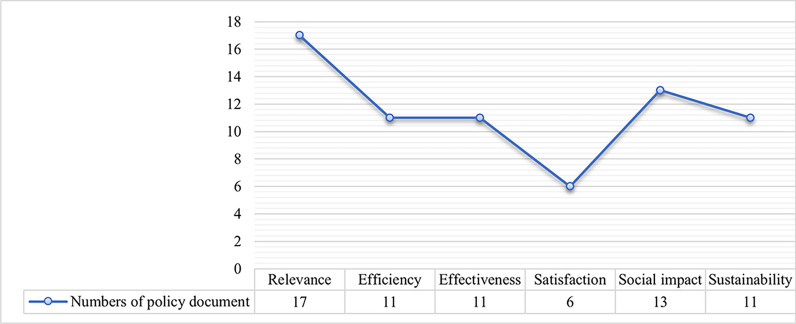
Line chart of ‘project performance objectives’ from the policy documents (*n* = number of policy documents)

The responsibilities of the purchasers were not only limited to finance and policy support (*n* = 22) but also included project supervision and evaluation (*n* = 19). The main responsibilities of the undertakers were mainly to implement old age care services, including assistance with daily life (*n* = 15) and health promotion services (*n* = 14), while the services such as respite care (*n* = 1) were rarely mentioned. The Ministry of Civil Affairs (MCA, *n* = 16), the National Health Commission (NHC, *n* = 11) and the Ministry of Finance (MF, *n* = 8) were most mentioned in the policy documents as the purchasers. The description of the undertakers in the policy documents was generalized as the social force (*n* = 17). In terms of the service objects, older adults with disability and dementia (*n* = 17), and the oldest old (*n* = 12) were paid more attention. See Fig. 4 for details. Moreover, project performance objectives were mentioned in all policy documents, among which the requirements for ‘satisfaction’, ‘efficiency’ and ‘effectiveness’ are less mentioned (Fig. 5).

### Deviation between policy formulation and project implementation

**Fig. 6:**
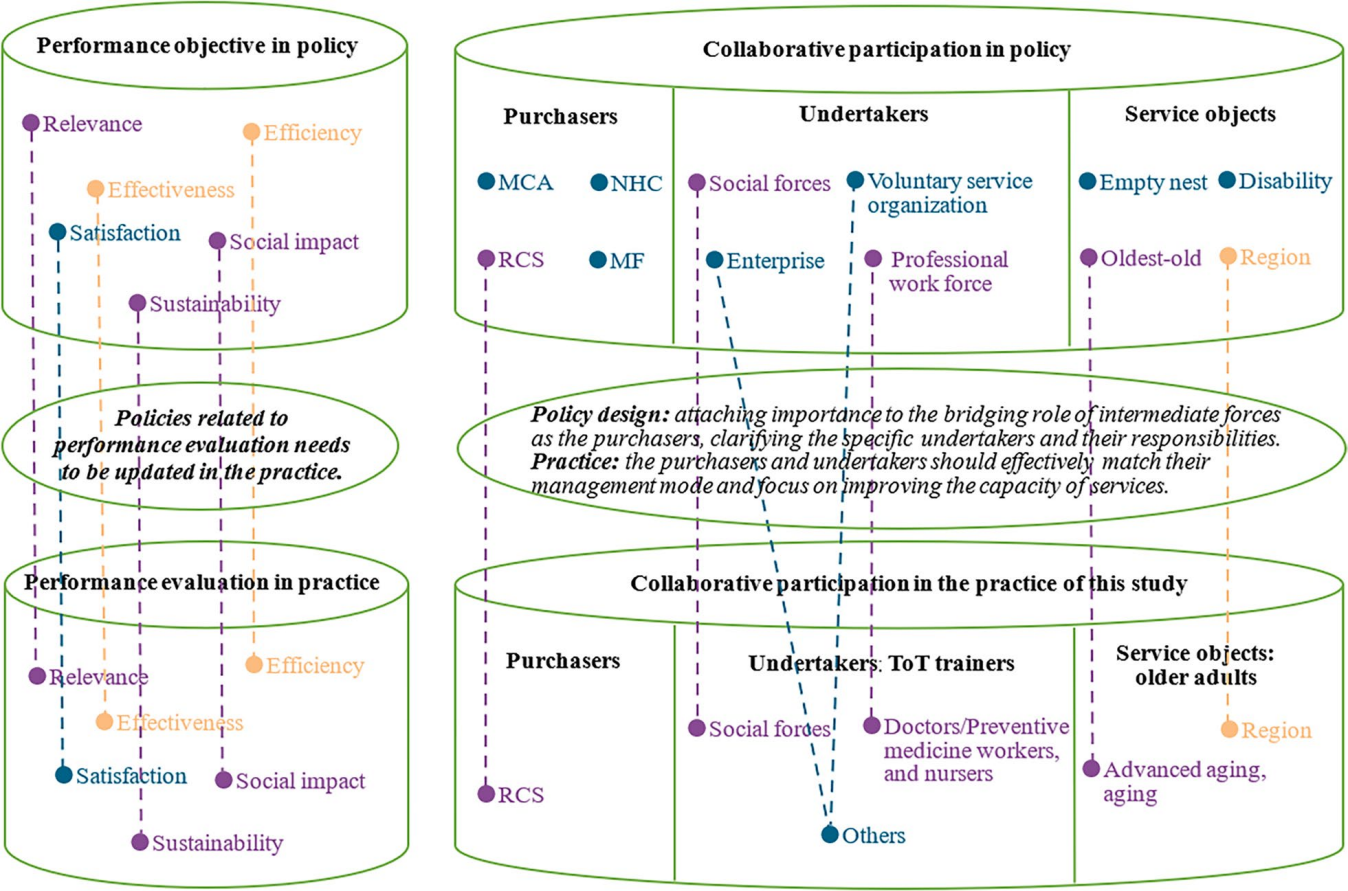
Deviation between policy design and implementation in practice. Purple dotted line: being equally important, valued or the same in both the policy design and practice; yellow dotted line: being equally unimportant, undervalued or little different in the policy design and practice; and blue dotted line: being opposite or cannot be compared in the policy design and practice

In terms of project performance, the efficiency, effectiveness, and sustainability requirements mentioned in the policy documents were almost similar to the results in the empirical study on performance evaluation. The satisfaction requirement was least mentioned in the included policy documents, while it was attached importance in the project of this study. In the empirical study, the project performance grades were different between different advanced ageing groups. As well, the policy documents also mentioned that the services focus should be tilted towards the oldest old. The Red Cross Society acts as the purchaser to purchase the services and ToT in the empirical study. However, from the included policy documents, this kind of relatively institutionalized and systematic social force was rarely mentioned. As for the description of undertakers, the policy design should clarify the specific undertakers and their responsibilities. Additionally, more opportunities should be given to various undertakers to better give full play to different professional abilities in practice (Table 4 and Fig. 6).

**Table 4:** Comparison of the results between cross sectional study and policy analysis

Dimension		Study design		
		Cross-sectional study	Policy documents analysis	Comparison
Project performance in practice/performance objective in the policy documents	Relevance (Mean ± standard deviation/*n*)	0.80 ± 0.22	17	+
	Efficiency	0.70 ± 0.24	11	−
	Effectiveness	0.78 ± 0.19	11	−
	Satisfaction	0.83 ± 0.20	6	×
	Social impact	0.81 ± 0.17	13	+
	Sustainability	0.80 ± 0.19	11	+
Factors associated with project performance/coordination of participants described in the policy documents	Service object	Advanced ageing (*P* < 0.05), ageing	Oldest old	+
		Region (southern, northern and central of Jiangsu)	Region (degree of economic hardship: rural and urban)	−
		Not measured	Disability, dementia	/
		Not measured	Lonely, empty nest, left behind	/
	Undertaker	Social worker	Social force	+
		Doctor/preventive medicine worker and nurse	Professional work force	+
		Others	Charitable organization, voluntary service organization, voluntary service organization of Red Cross Society	/
			Enterprise, industry association	/
		Training year (professional training)	−	/
	Purchaser	Red Cross Society	MCA, NHC, SC, MF, NDRC, RCSC, etc.	+

+ being equally important, valued or the same in both the policy design and practice; − being equally unimportant, undervalued or little different in the policy design and practice; × being opposite in the policy design and practice, / cannot be compared in the policy design and practice

## Discussion

### Performance of China’s local government purchase of social forces to participate in the old age care services

In this study, the group with poor performance only accounted for 17.0%, suggesting that the China’s local government purchase of social forces to participate in the old age care service projects had a good performance. It may be related to the fact that all old age care service projects in this study were supported by policies, resources and finance of the Red Cross Society of Jiangsu Branch, and the establishment of the ToT programme for the social forces to improve their professional skill. However, the deviation-standardized score of each dimension of performance suggested that efficiency needed to be improved. The imbalance of the input–output ratio could seriously affect the efficiency of the overall project [25]. Similar to enterprise organizations, operational efficiency is the determining element for them to formulate a certain business scope and scale. The key factor to maximize operational efficiency is the equal balance between the production possibility boundary (production content, capacity, etc.) and organizational possibility boundary (management, cost input, etc.) [26]. Therefore, for achieving the objective that efficient operation of the government purchase of old age care services, it is necessary to balance the relationship between the organizational possibility boundary of the purchasers and the undertakers and the production possibility boundary of the undertakers who were as the service producer.

Although the empirical projects included in the study were supported by the Red Cross Society of Jiangsu Branch with some organizational elements such as cost input and organizational management, while small social organizations may be limited by funding sources for a long time. Their inherent production possibility boundary such as content and capacity of production was difficult to cope with the expected organizational management objectives, which made it impossible for cost input to achieve efficient and effective service output. Additionally, some organizations put too much effort to deal with project assessment and ignored the improvement of actual effectiveness or ability of service production. They could not obtain high service output as well [27]. Moreover, some social service organizations, as service producers, were also managers. They lacked professional financial, organizational and other management personnel. Meanwhile, the project management mode developed by the purchaser puts forward vertical management requirements on the undertakers, which did not match the existing organizational management system and experience of the undertakers. All these lead to the balance between the organization possibility boundary and production possibility boundary cannot be matched, thus, the efficiency was difficult to achieve the best.

Therefore, the efficiency and effectiveness of the government purchase of old age care services can be improved from the above two aspects in the future. The purchasers should establish a reasonable project and fund supervision platform and adopt personalized budget and management mode to match the production possibility boundary of the undertakers. These could ensure the effectiveness of the organization and fund management input of the old age care services. At the same time, the undertakers should effectively improve their internal institutional setup, strengthen weak management abilities, eliminate redundant management and focus on improving the capacity of old age care services, so that they match their organizational possibility boundary and production possibility boundary to achieve the best marginal benefit.

### Factors of service objects and undertakers related to project performance and deviation from policy

The needs of older adults are more extensive and specialized with the deepening of advanced ageing [28], combining with the regional economic and social development, they can jointly affect the development of local old age care services. However, whether external social and economic factors will directly affect the performance of China’s local government purchase old age care services remains to be unknown. In this study, we compared the project performance grades between different service object groups. The results showed that the project performance grades were different with statistical significance between different advanced ageing groups, while the ageing groups were not, suggesting that the oldest old may be more prone to be disabled, dementia and other problems than the younger old with good physical functions and abilities of activities. They will be paid more attention to as the service object in old age care services [29]. Additionally, the policy documents in this field also mentioned that the services focus should be tilted towards older adults with disabilities, dementia and economic difficulties, or the oldest old. Therefore, the deeper the advanced ageing of the region, the more attention may be paid to the development of old age care services, and their performance may also change. From the perspective of public management, when the government purchased services, they usually considered the ‘demand side defect’ to measure the market demand for services. There is great demand for care for the special older population such as the oldest old, dementia and disability in China [30, 31].

It is worth noting that the region in the empirical study did not show similar results as the policy document. The performance grades between different regional groups were not statistically significant. The possible reason is that the regional division in the empirical study was only based on the socio-economic features of Jiangsu province. The degree of economic among different regions in Jiangsu province is still different from the rural–urban economic division that the policy document mentioned, in which more attention was proposed to be paid to the older adults in rural areas. In addition, there was no statistically significant difference in the project performance grades between the undertakers with different characteristics such as training years and professions, which may be related to the fact that the overall framework of training and project management did not change greatly every year. Thus, undertakers with different professional backgrounds could better grasp the key points of project content implementation and professional abilities after unified training. Therefore, the improvement of project performance may still need to focus on the internal factors and other closely related factors of project development, such as the project planning of social organizations, human resources, and their economic resources affected by external funding [32], so as to improve their production possibility boundary and organizational possibility boundary of old age care service to enhance the project performance grade.

### Responsibilities of purchasers and undertakers described in the policy documents and implementation in practice

The government purchase of services should be not only supported by finance, but should also require the joint efforts of multiple departments to formulate supporting policies, standards and specifications, and purchase contents. Additionally, multi-dimensional responsibility coordination of social forces should be established to form institutionalized and systematic process instruction of government purchase of social forces to participate in the old age care services. According to the results of the cooperative relationship between the policymaker departments and the analysis of the policy content, this aspect has been reflected. The multi-subject coordination pattern based on the local core was formed. Although the Red Cross Society of China and the China Disabled Persons’ Federation did not belong to departments of the State Council directly, they carried out or manage social services on a large scale. Their functions and responsibilities could serve as the main subject of communication between government departments and grassroots social organizations.

The policy document directly related to this empirical study on old age care services is the *Guiding Opinions on the Participation of the Red Cross Society in Old Age Care Services* [33]. The Red Cross Society of China, as the undertaker, strives for financial support from the government such as applying for financial funds and special government subsidies to improve financial security. Additionally, it acts as the purchaser to purchase the old age care services and ToT carried out by grassroots social forces such as society, professional institutions and universities. However, from the included policy documents, this kind of relatively institutionalized and systematic social forces (such as the Red Cross Society of China) were rarely mentioned in the government purchase of services. Therefore, the policy makers should pay more attention to the important bridging role of intermediate forces in service purchase to give more support to grassroots social forces.

As for the description of undertakers, although some policy documents proposed that voluntary service organizations, enterprises and industry associations should undertake the responsibility of old age care services, in many cases they were described by the generalized term ‘social force’. The policy design should be more standardized and clarify the specific undertakers purchased by the government. It will be helpful to clarify their purchase boundary in the process of purchasing old age care services, that is, which old age care services could be purchased, and whether any undertakers are matching these specific contents, responsibilities and functions. All of these are vital for improving the unclear boundary scope, weak operability and undertakers’ ambiguous responsibilities and functions in the existing practice. The purchase boundary mentioned in the existing policy documents mainly focussed on the daily life service, health promotion or management and social participation of older adults, which could reflect the concept of ‘combination of medical care’ to a certain extent. However, the special service content of the ‘medical’, such as respite car,e was relatively less mentioned in the policy documents. So, they did the same with the social forces such as professional industry associations, who have the professional ability to undertake or direct these special services. Therefore, before formulating the policy in this field, technology such as information platforms and big data analysis should be adopted to effectively identify the needs of service objects, clarify the purchase boundary, balance the service structure of the ‘combination of medical care’ and avoid the absence of demand identification.

### Project performance objectives described in the policy documents and expectation in bridging the deviation between policy formulation and implementation

In the policy document analysis, the proposal of project performance objectives in the policy was mainly presented in the form of the basic principles of the project or the expected achievement objectives. The method of output process for the performance outcome, that is, the specific evaluation process or evaluation scheme was rarely mentioned. The policy document *the Guiding Opinions on the Red Cross Society's Participation in Old Age Care Services*, which is directly related to the empirical study, did not put forward the specific performance objective’s evaluation scheme at the beginning as well [33]. However, during the implementation of this project, the Red Cross Society of Jiangsu Branch gradually improved the formulation of the project performance evaluation scheme [13]. In most policy documents such as pilot projects, opinions or notifications, the policymaking departments may only put forward development opinions or suggestions in the general direction on the performance evaluation. The formulation of the detailed content scheme containing a generalized and systematic working system needs to be formed in the process of practice. It also suggests the requirement of periodic update of policy documents to continuously optimize and improve the early policy scheme to provide specific guidance for the work.

In addition, the satisfaction requirement was least mentioned in the included policy documents. Although it was rarely mentioned with explicit words in the policy documents, satisfaction as a direct reflection of the service object’s evaluation of the effect of the project, therefore, was attached importance in the project of China’s local government purchase of old age care services of this study. It may be the reason for the high mean score of this dimension in the performance evaluation of the empirical study. Efficiency, effectiveness and sustainability requirements were mentioned in less than half of the policy documents, which were almost similar to the results of the empirical study on performance evaluation. Therefore, when the policymaking departments design policy, they should pay more attention to the efficiency and effectiveness of project implementation and emphasize the improvement of the project supervision and evaluation mechanism to promote ‘progress’ by ‘evaluation’. It will ensure the real implementation of the project by external thrust rather than formal implementation and bridge the gap between policy formulation and policy implementation.

### Limitations

There are some limitations to this study. Firstly, in the quantitative study, the accessibility of service objects data such as the degree of ageing and advanced ageing in the China’s local government purchase of aged service projects was limited. The data of the service objects obtained by the local statistical report may not accurately and directly reflect their overall characteristics. It suggested that the undertakers and the purchasers should improve the basic information of the service object in the future to provide a more accurate data basis for the performance evaluation research. Additionally, a national wide evaluation on performance of government purchase of old age care service projects is also essential. Additionally, we only included the national-level policy documents in the study. Provincial policy documents in this field can be included in later research to analyse regional differences.

## Conclusion

Our study stands out for its innovative approach, which delves into the policy implementation deviation. Compared with opinion research, the multi methods research we conducted could provide more robust and comprehensive evidence in this field. Furthermore, similar to views in the Smith Policy Implementation Model, factors such as the policy itself, the skills and capabilities of the policy implementation agencies, and the external environment should be considered during the policy implementation process. According to the results of the empirical study and policy document analysis in the field of government purchase of old age care services, the policy design should give more consideration to the responsibilities and functions of the intermediate purchasing force to give full play to their role as a bridge between government departments and grassroots social forces. The description of the undertakers and their responsibilities at the old age care services should be more targeted. In practice, the purchasers and undertakers need to follow relevant regulations and systems specified in the policy documents, effectively improve weak management abilities, eliminate redundant management,and focus on improving the capacity of old age care services and their close factors. It will be beneficial for matching the organizational possibility boundary and production possibility boundary to achieve the best marginal benefit. In addition, the performance evaluation scheme should be gradually improved and periodically updated in the practice process to bridge the deviation between policy implementation and policy formulation. Our findings help in identifying the deviation between policy and implementation and promote the systematic uptake of policy into routine practice and, hence, to improve the quality and effectiveness of government purchase of old age care services. In other fields, future studies should also focus on the deviation between policy design and implementation instead of sole policy design, which could promote the effective implementation of the policy in practice.

### Supplementary Information

**Additional file 1. **Policy documents included in the analysis

## Data Availability

The datasets used and/or analysed during the current study are available from the corresponding author on reasonable request.

## Abbreviations

ToT: Training of Trainer
SCVTP: Senior Care Volunteers Training Program
MCA: Ministry of Civil Affairs
NHC: National Health Commission
SC: State Council
CCCYL: Central Committee of the Communist Youth League
NDRC: National Development and Reform Commission
MT: Ministry of Transport
ME: Ministry of Education
MJ: Ministry of Justice
SAMR: State Administration for Market Regulation
GOCCCPC: General Office of Central Committee of the Communist Party of China
MCT: Ministry of Culture and Tourism
MPS: Ministry of Public Security
NRA: National Railway Administration
MC: Ministry of Commerce
MHRSS: Ministry of Human Resources and Social Security
MHURD: Ministry of Housing and Urban–Rural Development
MF: Ministry of Finance
NRRA: National Rural Revitalization Administration
RCSC: Red Cross Society of China
CBIRC: China Banking and Insurance Regulatory Commission
CDPF: China Disabled Persons’ Federation
NHSA: National Healthcare Security Administration
MNR: Ministry of Natural Resources
MEE: Ministry of Ecology and Environment
PBC: People’s Bank of China
CAAC: Civil Aviation Administration of China
SPB: State Post Bureau
GASC: General Administration of Sport of China
